# The Surgical Section of the College of Physicians of Philadelphia

**Published:** 1894-06

**Authors:** John B. Roberts

**Affiliations:** Chairman, in the chair


### THE SURGICAL SECTION OF THE COLLEGE OF PHYSICIANS OF PHILADELPHIA.

MEETING OF MARCH 9, 1894.

*Dr. John B. Roberts, Chairman, in the chair.*

DR. J. B. ROBERTS read a paper on "Operation for Ancient Dislocation of Elbow and Bridge of Callus between Radius and Ulna." (See page 299).

DR. T. G. MORTON also read a paper on "Cases of Delayed Union in Fractures." (See page 303.)

DISCUSSION.

DR. G. G. DAVIS: I think this is a good example of how plaster dressings do not keep a limb quiet. Inasmuch as they are used for that specific purpose, I think it is worth while to call attention to the fact that at least in the way they are often applied, with a layer of cotton beneath, they are liable to permit motion at the seat of fracture. Of course, this man had some dressing beneath the plaster bandage, which was probably the cause of its getting loose. I should like to ask Dr. Morton whether in case of delayed union he keeps the parts absolutely quiet, or whether he considers it is best to allow some motion. From the cases reported it is evident that they can be cured both by permitting motion and by keeping them at rest.

DR. MORTON: I would keep them absolutely quiet until the probability of repair through quiet seems doubtful, then I would put the limb into a movable apparatus.

DR. DAVIS: That is the question I wished to call attention to: the first man was treated with an apparatus which did not keep the limb quiet, then it was put at rest and union occurred, and in the second, the opposite was the case. I think many of the cases of delayed union are kept so from lack of proper rest. I have seen several of that character.

RUBBER-TISSUE GLOVES FOR PROTECTING THE HANDS DURING OPERATIONS, ETC.

DR. T. S. K. MORTON called the attention of the Section to thin rubber gloves, which have been in the market for some time and are coming into use for general surgical purposes and for handling strong solutions. "I have found the rubber-tissue gloves extremely useful in handling offensive cases. With them it becomes a pleasure to make rectal examinations, because the skin of the hands does not become saturated with fetor, and it is wonderful how many more examinations one makes. Also in handling strong solutions, or even in operating upon septic cases they have an excellent field. The rubber is so very thin that it interferes very little with the tactile sense. As a rule, they go on with great ease and come off readily. The dealer said he was not sure that they would last very long in handling instruments, but thus far I have used them considerably and they are still practically in perfect order. But if they could be used only for a few times, the price, $2.50, with forty per cent. discount to hospitals, is not excessive. I think them of great value when handling morbid growths, or making post-mortems, where it is possible to be inoculated. They bear steam sterilization and soaking in strong solutions of carbolic acid or bichloride."

